# Coffee intake one hour prior to phlebotomy produces no clinically significant changes in routine biochemical test results

**DOI:** 10.11613/BM.2023.020705

**Published:** 2023-06-15

**Authors:** Silvia F Benozzi, Gisela Unger, Amparo Campion, Pablo G Milano, Graciela L Pennacchiotti

**Affiliations:** 1 Departamento de Biología, Bioquímica y Farmacia, Universidad Nacional del Sur (UNS), Bahía Blanca, Argentina; 2 Hospital Municipal de Agudos "Dr. Leónidas Lucero", Bahía Blanca, Argentina; 3 Instituto de Investigaciones Bioquímicas de Bahía Blanca (INIBIBB), Consejo Nacional de Investigaciones Científicas y Técnicas (CONICET), Universidad Nacional del Sur (UNS), Bahía Blanca, Argentina

**Keywords:** fasting, coffee intake, blood sample collection, preanalytical variability, biochemical tests

## Abstract

**Introduction:**

Although current guidelines recommend not drinking coffee prior to phlebotomy, our hypothesis is that drinking coffee does not affect the clinical interpretation of biochemical and haematological test results.

**Materials and methods:**

Twenty-seven volunteers were studied in basal state (T0) and 1h after (T1) drinking coffee. Routine haematological (Sysmex-XN1000 analyser) and biochemistry parameters (Vitros 4600 analyser) were studied. Results were compared using the Wilcoxon test (P < 0.05). A clinical change was considered when mean percent difference (MD%) was higher than the reference change value (RCV).

**Results:**

Coffee intake produced statistically, but not clinically, significant: i) increases in haemoglobin (P = 0.009), mean cell haemoglobin concentration (P = 0.044), neutrophils (P = 0.001), albumin (P = 0.001), total protein (P = 0.000), cholesterol (P = 0.025), high density lipoprotein cholesterol (P = 0.007), uric acid (P = 0.011), calcium (P = 0.001), potassium (P = 0.010), aspartate aminotransferase (P = 0.001), amylase (P = 0.026), and lactate dehydrogenase (P = 0.001), and ii) decreases in mean cell volume (P = 0.002), red cell distribution width (P = 0.001), eosinophils (P = 0.002), and lymphocytes (P = 0.001), creatinine (P = 0.001), total bilirubin (P = 0.012), phosphorus (P = 0.001), magnesium (P = 0.007), and chloride (P = 0.001).

**Conclusion:**

Drinking a cup of coffee 1 hour prior to phlebotomy produces no clinically significant changes in routine biochemical and haematological test results.

## Introduction

Current patient preparation guidelines for phlebotomy recommend, among other measures, that patients should go to the laboratory between 7:00 a.m. and 9:00 a.m. with a 12 h fasting period and that, although they are allowed to drink water during this period, they should abstain from drinking alcohol during the previous 24 hours (*1*). They also indicate that on the morning of blood collection they should neither smoke nor drink caffeine-containing beverages, nor chew gum (*1*). Although a previous joint recommendation for venous blood sampling, issued by the European Federation of Clinical Chemistry and Laboratory Medicine (EFLM) Working Group for Preanalytical Phase (WG-PRE) and the Latin American Working Group for Preanalytical Phase (WG-PRE-LATAM) of the Latin America Confederation of Clinical Biochemistry (COLABIOCLI), admits that fasting may pose some logistical difficulties, non-fasting blood collection is permitted only in cases of emergencies or for parameters for which there is evidence that fasting is not required (*1*). The recommendation to abstain from drinking caffeine-containing beverages prior to phlebotomy is supported by previous findings according to which caffeine, which is present in many infusions and soft drinks (coffee, tea or cola-type soft drinks), may affect various analytes, such as blood glucose concentration (Glc), non-esterified fatty acids, and ionized calcium concentrations, all of which increase after caffeine intake (*2*-*4*). Results from previous studies on the influence that the intake of a standardized breakfast including a caffeine-containing beverage has on laboratory parameters are ambiguous (*5*, *6*).

It is known that 1.6 billion cups of coffee are consumed per day (*7*). Coffee, the second most widely consumed beverage in the world after water, is a complex mixture composed of more than 1000 chemical components (*7*). Caffeine and chlorogenic acids are the most abundant chemical components of coffee which also contains micronutrients, such as magnesium, potassium, vitamin E, niacin, and the diterpenes cafestol and kahweol, the latter of which has been reported as responsible for increasing cholesterol (*7*, *8*).

Caffeine, one of the most abundant components of coffee, is both rapidly and completely absorbed in humans, 99% being, in fact, absorbed within 45 minutes after intake. Maximal plasma concentrations occur between 15 and 120 minutes after oral intake, and they may be influenced by the administration route, administration form or other components of the diet. Once absorbed, caffeine is distributed throughout body water (*4*).

The clinical impact of drinking coffee prior to phlebotomy is still not known. The purpose of the present work was therefore to study whether clinically significant changes occur in routine biochemical test results after drinking a cup of coffee without additives 1 h before phlebotomy, based on the hypothesis that this does not affect the clinical interpretation of results.

## Materials and methods

### Study design

This is an experimental longitudinal study carried out in a laboratory at Hospital Municipal de Agudos "Dr. Leónidas Lucero" in Bahía Blanca city, province of Buenos Aires, Republic of Argentina, during July, 2022.

### Subjects

The study population in the present work included 27 volunteers (20 women and 7 men), with a median age of 24 years (min-max: 22-33 years), who reported having no previous pathologies and who, according to their self-perception, were “healthy”. They also reported in a questionnaire on their consumption habits that they were neither taking any medication or vitamin supplements nor consuming natural herbs or their derivatives on a regular basis.

### Methods

The study protocol was approved by the Bioethics Committee of Hospital Municipal de Agudos "Dr. Leonidas Lucero". All volunteers were informed on the nature and procedures of this study and gave their written informed consent to participate in the present work.

Standardized guidelines were followed during the procedure of preparation and blood sample collection (*1*, *9*). In Argentina, open blood collection systems - in which a syringe is combined with a needle - are used under strict biosecurity standards (*10*). In order to detect any preanalytical error that may occur during blood collection through the open system, sample haemolysis was assessed via the haemolysis index. Samples were not haemolysed.

A study protocol previously employed to study the effect of preanalytical variables on laboratory test results was used (*6*, *11*, *12*). Volunteers attended the laboratory between 7:00 and 9:00 a.m. after a 12 h fasting period for the first phlebotomy (T_0_). After this, each volunteer drank a 90 mL cup of coffee prepared in an automatic coffee machine using espresso capsules provided by a commercial brand. The espresso capsules contained 5.5 g of coffee, 60 mg of caffeine, and had neither sugar nor other additives. After coffee intake, volunteers remained seated and at rest for 1 h until the second phlebotomy (T_1_) was performed.

Samples were collected via antecubital vein puncture using needles 21Gx1 (NEOJET, Zhejiang Ougian Medical Apparatus Co, Wenzhou, China) and 10 mL syringes (Hongda, Jiangxi Hongda Medical Equipment Group Ltd., Nanchang, China). After blood sample collection, samples were distributed in tubes with K3EDTA for haematological determinations and in tubes with lithium heparin (both tubes were purchased from Tecnon, Laboratorios Argentinos, Berisso, Argentina) for clinical chemistry analyte measurements. The biochemical tests were performed immediately afterwards. The haematological parameters were measured using an haematological Sysmex XN 1000 analyser (Sysmex, Kobe, Japan), and the clinical chemistry tests were performed using a Vitros 4600 autoanalyser (Ortho Clinical Diagnostics, New Jersey, USA). Manufacturers provided reagents, calibrators, and controls. The methodological principles of the clinical chemistry tests performed are shown in Table 1. The analytical coefficients of variation (CV_A_), which were calculated on a monthly basis for each analyte, are shown in Tables 2 and 3.

**Table 1:** Methodological principles applied for the measurement of biochemistry analytes

**Analyte**	**Method**
Glc	Colorimetric/enzymatic method using glucose oxidase
Alb	Colorimetric method using bromocresol green
TP	Colorimetric method using copper tartrate
Urea	Colorimetric/enzymatic method using urease and ammonia indicator
CREA	Enzymatic method using creatinine amidohydrolase, creatine amidinohydrolase, sarcosine oxidase and peroxidase
TBIL	Diazo colorimetric method
CHOL	Colorimetric/enzymatic method using leuco dye
HDL	Direct/colorimetric method using leuco dye
LDL	Direct/colorimetric method using quinone dye
Tg	Colorimetric/enzymatic method using leuco dye
UA	Colorimetric/enzymatic method using uricase
GGT	Multiple-point rate kinetic/UV method (37°C)
ALP	Multiple-point rate kinetic/UV method (37°C)
AST	Multiple-point rate kinetic/UV method (37°C)
ALT	Multiple-point rate kinetic/UV method (37°C)
AMY	Two-point rate kinetic/UV method (37°C)
LIP	Two-point rate kinetic/UV method (37°C)
LD	Multiple-point rate kinetic/UV method (37°C)
CK	Multiple-point rate kinetic/UV method (37°C)
Ca	Colorimetric method using arsenazo III dye
Phos	Colorimetric method using p-methylaminophenol sulphate
Mg	Colorimetric method using formazan dye derivative
Na	Direct potentiometry
K	Direct potentiometry
Cl	Direct potentiometry
Glc - glucose. Alb - albumin. TP- total proteins. CREA - creatinine. TBIL - total bilirubin. CHOL - total cholesterol. HDL - high density lipoprotein cholesterol. LDL - low density lipoprotein cholesterol. Tg - triglycerides. UA - uric acid. GGT - gamma-glutamyltransferase. ALP - alkaline phosphatase. AST - aspartate aminotransferase. ALT - alanine aminotransferase. AMY - amylase. LIP - lipase. LD - lactate dehydrogenase. CK - creatine kinase. Ca - calcium. Phos - inorganic phosphate. Mg - magnesium. Na -sodium. K - potassium. Cl - chloride.	

**Table 2:** Variations in routine haematological parameters 1 h after coffee intake

**Parameter (unit)**	**CV_A_ (%)**	**CV_I_ (%)**	**T_0_** **(N = 27)**	**T_1_** **(N = 27)**	**P**	**MD%**	**RCV (%)**
RBC (x10^12^/L)	0.6	2.6	4.6 (4.4-5.0)	4.6 (4.5-5.0)	0.063	0.9	7.4
Hb (g/L)	0.6	2.7	134 (131-145)	138 (132-149)	0.009	1.1	7.7
Hct (L/L)	0.9	2.8	0.40 (0.40-0.44)	0.41 (0.40-0.44)	0.222	0.7	8.2
MCV (fL)	0.7	0.8	88.8 (86.8-91.0)	88.7 (86.2-90.8)	0.002	- 0.2	2.9
MCH (pg)	0.7	0.8	29.6 (28.8-30.3)	29.7 (28.8-30.3)	0.088	0.2	2.9
MCHC (g/L)	0.7	1.0	332 (330-336)	336 (329-338)	0.044	0.4	3.5
RDW-SD (fL)	1.0	1.7	39.4 (37.7-40.7)	39.0 (37.3-40.5)	0.001	- 0.8	5.5
WBC (x10^9^/L)	1.7	10.8	6.60 (6.0-7.1)	6.60 (5.4-7.3)	0.718	1.2	30.3
NEU (x10^9^/L)	2.6	14.0	3.15 (2.55-3.64)	3.57 (2.64-4.48)	0.001	19.7	39.5
EOS (x10^9^/L)	6.4	15.0	0.10 (0.08-0.17)	0.10 (0.07-0.15)	0.002	- 15.8	45.2
BASO (x10^9^/L)	2.9	12.4	0.03 (0.02-0.04)	0.03 (0.02-0.04)	0.387	20.0	35.3
LYMP (x10^9^/L)	3.9	10.8	2.51 (1.93-3.19)	1.98 (1.66-2.32)	0.001	- 18.2	31.8
MONO (x10^9^/L)	6.9	13.3	0.48 (0.39-0.60)	0.51 (0.43-0.61)	0.931	1.4	41.5
Plt (x10^9^/L)	3.4	7.6	264 (228-307)	271 (233-308)	0.221	1.2	23.1
MPV (fL)	1.3	2.3	10.5 (9.7-10.9)	10.5 (9.7-11.0)	0.529	- 0.4	7.3
Values are presented as median and interquartile range. CV_A_ - analytical coefficient of variation. CV_I_ - within-subject biological variation. T_0_ - basal state. T_1_ - 1 h after coffee intake. MD% - mean percent difference 1 h after coffee intake. RCV - reference change value. P value indicates significance by Wilcoxon Signed Rank Test for paired samples. P < 0.05 was considered statistically significant. RBC - red blood cells. Hb - haemoglobin. Hct - haematocrit. MCV - mean cell volume. MCH - mean cell haemoglobin. MCHC - mean cell haemoglobin concentration. RDW-SD - red cell distribution width standard deviation. WBC - white blood cell. NEU - neutrophils. LYMP - lymphocytes. MONO - monocytes. EOS - eosinophils. BASO - basophils. Plt - platelet count. MPV - mean platelet volume.							

**Table 3:** Variations in clinical chemistry analytes 1h after coffee intake

**Analyte (unit)**	**CV_A_ (%)**	**CV_I_ (%)**	**T_0_** **(N = 27)**	**T_1_** **(N = 27)**	**P**	**MD%**	**RCV (%)**
Glc (mmol/L)	1.9	5.0	4.8 (4.5-5.1)	4.7 (4.4-5.0)	0.165	- 1.3	14.8
Alb (g/L)	2.3	2.5	42 (41-44)	43 (42-46)	0.001	3.3	9.4
TP (g/L)	1.8	2.6	72 (69-75)	74 (70-76)	0.001	3.0	8.8
Urea (mmol/L)	1.9	13.9	4.3 (3.7-4.7)	4.2 (3.7-4.7)	0.244	- 0.2	38.9
CREA (µmol/L)	1.2	4.5	73 (65-86)	70 (63-82)	0.001	- 4.8	12.9
TBIL (µmol/L)	2.5	20.0	10 (5-21)	9 (7-21)	0.012	- 6.7	55.9
CHOL (mmol/L)	2.1	5.3	4.6 (4.0-5.0)	4.6 (4.3-5.1)	0.025	1.6	15.8
HDL (mmol/L)	4.7	5.8	1.9 (1.4-2.3)	1.9 (1.4-2.3)	0.007	3.0	20.7
TG (mmol/L)	4.6	20.0	1.0 (0.6-1.3)	1.0 (0.7-1.3)	0.367	- 2.3	56.9
UA (µmol/L)	1.1	8.3	262 (244-303)	262 (250-303)	0.011	0.6	23.2
GGT (U/L)	1.5	8.8	18 (16-23)	18 (15-23)	0.296	1.5	25.6
ALP (U/L)	6.4	5.3	52 (44-62)	52 (44-63)	0.208	1.4	23.0
AST (U/L)	2.9	9.6	21 (20-24)	22 (21-26)	0.001	4.0	27.8
ALT (U/L)	5.4	10.1	25 (21-30)	25 (21-28)	0.333	0.4	31.7
AMY (U/L)	3.3	6.6	69 (64-84)	73 (66-89)	0.026	3.5	20.5
LIP (U/L)	0.9	9.2	81 (67-103)	80 (63-96)	0.501	- 0.8	25.6
LD (U/L)	1.3	5.2	369 (341-420)	382 (356-416)	0.001	5.3	14.9
CK (U/L)	4.3	15.0	99 (72-125)	98 (75-121)	0.330	0.7	43.3
Ca (mmol/L)	1.3	1.8	2.34 (2.30-2.45)	2.40 (2.34-2.47)	0.001	1.5	6.2
Phos (mmol/L)	1.3	7.8	1.23 (1.29-1.26)	1.16 (1.00-1.23)	0.001	- 8.1	21.9
Mg (mmol/L)	1.2	2.9	0.82 (0.78-0.82)	0.82 (0.74-0.86)	0.007	1.8	8.7
Na (mmol/L)	1.3	0.5	139 (139-141)	140 (138-141)	0.135	0.2	3.9
K (mmol/L)	1.9	4.1	4.3 (4.1-4.4)	4.4 (4.3-4.5)	0.010	3.5	12.5
Cl (mmol/L)	1.8	1.1	105 (104-106)	104 (103-106)	0.001	0.7	5.8
Values are presented as median and interquartile range. CV_A_ - analytical coefficient of variation. CV_I_ - within-subject biological variation. T_0_ - basal state. T_1_ - 1 h after coffee intake. RCV - reference change value. MD% - mean percent difference 1 h after coffee intake. P value indicates significance by Wilcoxon Signed Rank Test for paired samples. P < 0.05 was considered statistically significant. Gl c- glucose. Alb - albumin. TP - total protein. CREA - creatinine. TBIL - total bilirubin. CHOL - total cholesterol. HDL - high-density lipoprotein cholesterol. LDL - low-density lipoprotein cholesterol. Tg - triglycerides. UA - uric acid. GGT - gamma-glutamyltransferase. ALP - alkaline phosphatase. AST - aspartate aminotransferase. ALT - alanine aminotransferase. AMY - amylase. LIP - lipase. LD - lactate dehydrogenase. CK - creatine kinase. Ca - calcium. Phos - inorganic phosphate. Mg - magnesium. Na - sodium. K - potassium. Cl - chloride.							

### Statistical analysis

As the present study was performed in a group composed of 27 subjects (N < 30), data analysis was carried out using non-parametric statistics. Data were expressed as median and interquartile range. The comparison of results between T_0_ and T_1_ was made with the non-parametric Wilcoxon Signed Rank Test for paired samples (*13*). Statistical Package for Social Science for Windows (SPSS) software (Version 15.0. Chicago, IL, USA) was used for data analysis. Statistical significance was set at P < 0.05.

The clinically significant change for each analyte was calculated using the following reference change value (RCV) equation: RCV (%) = 2^1/2^ x Z x (CV_I_^2^ + CV_A_^2^)^1/2^; where *Z* = 1.96; CV_I_ is the within-subject biological variation coefficient obtained from EFLM database; and CV_A_ is the analytical coefficient of variation from the laboratory internal quality control (*14*-*19*). The mean percent difference (MD%) between T_0_ and T_1_ for each parameter was compared with its respective RCV. Mean percent difference was calculated using the following formula:



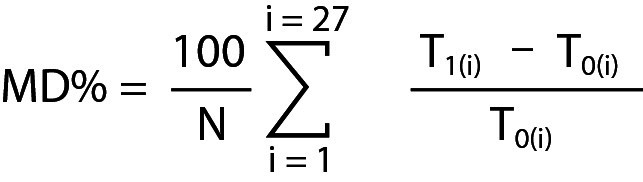



where N = 27 (total number of volunteers that participated in the study), T_1(i)_ is the value of the parameter in the blood sample of each individual 1 h after the intake of a cup of coffee (T_1_), T_0(i)_ is the value of the parameter in the blood sample of each individual at basal time (T_0_), and i means individual (*9*, *12*, *20*). A clinically significant change was considered to have occurred after coffee intake when MD% was greater than the RCV.

## Results

The results collected from the biochemical tests performed after coffee intake are shown in Tables 1 and 2. Table 1 indicates that the following haematological parameters were higher 1 h after drinking coffee: haemoglobin (Hb), mean cell haemoglobin concentration (MCHC), and neutrophils (NEU). In contrast, the following parameters were lower at T_1_ than at T_0_: mean cell volume (MCV), red cell distribution width standard deviation (RDW-SD), eosinophils (EOS), and lymphocytes (LYMP). The statistically significant variations observed in the haematological parameters analysed were not clinically relevant 1 h after coffee intake.

The results collected from the clinical chemistry tests performed are shown in Table 2. Values were found to be higher at T_1_ than at T_0_ in the concentrations of albumin (Alb), total protein (TP), total cholesterol (CHOL), high-density lipoprotein cholesterol (HDL), uric acid (UA), calcium (Ca), potassium (K) and enzyme activities of aspartate aminotransferase (AST), amylase (AMY), and lactate dehydrogenase (LD). After coffee intake, the concentrations of the following parameters were lower: creatinine (CREA), total bilirubin (TBIL), inorganic phosphate (Phos), magnesium (Mg), and chloride (Cl). However, none of the above-listed variations had clinical significance because MD% was lower than the RCV.

## Discussion

Although none of the haematological parameters analysed in this study showed clinically significant changes 1 h after coffee intake, some of them did have statistically significant changes, namely NEU, EOS, and LYMP. After coffee intake, NEU count was higher (19.7%) than at T_0_. Previous research conducted by our group on the influence of a standardized breakfast intake (consisting of a cup of cappuccino coffee containing 75 mg of caffeine and 4 sweet cookies) on routine biochemical test results revealed a similar increase (16.4%) in NEU count. In this previous study, blood samples had also been collected 1 h after breakfast (*6*). Likewise, further research from our group also showed an increase in NEU count (11.9%) after mate intake (*21*). Mate is a popular traditional beverage usually drunk at breakfast in some South American countries and is prepared with yerba mate (*Ilex paraguariensis* St. Hil.), which - among its components - contains caffeine (*22*).

As for the EOS count values recorded in the present study, they were lower at T_1_ (- 15.8%) than at T_0_. This coincides with the EOS count values observed: i) 1 h after drinking 300 mL of water (- 12.1%), ii) 1 h after having a standardized breakfast that included coffee (- 22.1%), and iii) 1 h after drinking mate (- 29%), although in none of these cases did the MD% exceed the RCV (*6*, *12*, *13*, *21*, *22*). As for the LYMP count values, they were lower (- 18.2%) at T_1_ than at T_0_. This was also observed in the LYMP count values recorded in previous studies from our group which analysed the influence of: i) 300 mL water intake (- 24%), ii) breakfast intake (- 29.1%), and iii) mate intake (- 26.3%) before phlebotomy (*6*, *12*, *21*). Although all these variations were statistically significant, they were not clinically relevant. The changes observed in the cell counts, all without clinical relevance and with values which remained within the reference intervals, could be attributed not to coffee intake but to the cortisol-regulated circadian rhythm affecting immune system cells and therefore to the sampling time (between 8:00 and 10:00 a.m.) (*23*, *24*).

Although the results of the clinical chemistry tests performed in the present work did not show any relevant changes, some considerations are worth mentioning. For example, previous studies have demonstrated that coffee intake produces an acute increase in Glc. Blood Glc under fasting conditions increases by almost 12% within 1 h after consuming a 12 oz. cup of coffee with milk (approximately 350 mL) (*3*, *25*). A recent study from our group revealed an increase of 7.5% in the concentration of Glc 1 h after a standardized breakfast intake consisting of a cup of cappuccino coffee with 4 sweet cookies. Still, this increase was not clinically significant (*6*).

The values recorded for TP (3.0%), CHOL (1.6%), UA (0.6%), AST (4.0%), and LD (5.3%) were higher at T_1_ compared to those at T_0_. This is in agreement with the values recorded in a previous study from our group after a 300 mL water intake (2.2%, 5.2%,1.2%, 2.2%, 9.2%, and 5.3%, respectively) (*9*). We also observed that TP (2.8%), Alb (1.6%), UA (1.6%), and AST activity (3.1%) values were higher after a standardized breakfast intake than at T_0_ (*6*). The higher concentration of UA with respect to the basal value has been attributed to the blood sampling collection time as a consequence of the diurnal rhythm of this analyte (*5*).

As for CREA, the values (- 4.8%) recorded after coffee intake were lower than those at T_0_. This was also observed in previous studies after a standardized breakfast intake (- 1.9%) and after mate intake (- 5.2%) (*6*, *21*). The TBIL concentration values were also lower (- 6.7%) with respect to those at the basal state. This variation has been attributed to the blood sampling collection time because of the diurnal rhythm of this analyte (*5*).

The effect of caffeine on lipids is controversial and it depends on the method of coffee preparation. Previous research claims that diterpernes in coffee, such as cafestol and kahweol, whose concentration depends on how coffee is prepared, are responsible for increasing CHOL (*8*). An hour after coffee intake, CHOL concentration was found to be 1.6% higher than that under fasting conditions. This was similar to the increase in CHOL concentration (1.2%) recorded after 300 mL water intake (*11*). As for HDL concentration, it was of 3.0% at T_1_. In the two cases, the variations observed had no clinical significance and remained within the reference range.

The higher AMY activity recorded after coffee intake (3.5%) in the present work does not coincide with the data collected by Plumelle *et al.* showing no significant changes in the levels of this enzyme after a standardized breakfast containing either tea or coffee (*5*). In any case, in the present study the variation observed was not clinically significant.

On the other hand, previous research has shown that caffeine increases the concentration of ionized Ca by releasing Ca from intracellular deposits (*4*). In the present study, although total Ca was found to be 1.5% higher at T_0_ than at T_1_, it did not exceed the upper reference limit and the observed change had no clinical significance.

Phosphate concentration was observed to decrease by 8.1%. This coincides with results collected by Plumelle *et al.* who reported a decrease of 8% in this electrolyte after a standardized breakfast intake (*5*). This change was attributed to the blood sampling collection time because of Phos diurnal ryhthm (*5*). Although previous research has reported that coffee contains Mg and K, the increase observed in the present study in the level of 1.8% for Mg and 3.5% for K was not clinically significant (*7*).

The limitations of our study include: the low number of participants, gender- and age-bias, and the fact that the samples analysed were collected only from “apparently healthy” volunteers. Patient populations differ in their diets as well as in their dietary behaviours because of their cultural habits. This impacts the concentrations of the different biochemical constituents that are routinely measured in the laboratory (*26*, *27*). On the other hand, as the focus of attention of the present work was on the changes occurring in the biochemical test results collected 1 h immediately after coffee (90 mL) intake, its findings cannot be extrapolated to other phlebotomy schedules because the concentrations of several analytes depend on circadian rhythms and the effect of larger coffee intakes is unknown.

Caffeine content depends on i) bean varieties, sources, and harvesting methods; ii) bean batches; iii) roasting procedures; and iv) milling conditions. Furthermore, not all coffee varieties are the same as they depend on the commercial brand. In addition, coffee preparation/boiling may affect its chemical composition (*8*). Previous research has reported that caffeine content *per* cup of coffee varies among commercial brands from 51 to 322 mg. In this respect, the Farm Security Administration (FSA) has reported that an average cup of coffee contains 140 mg of caffeine. Guideline figures from the International Food Council in the United States suggest that an 8-oz (~225 mL) cup of instant coffee contains 60-85 mg of caffeine and that a 1 oz. (~28 mL) cup of espresso coffee contains 30-50 mg of caffeine (*28*).

Although all the above must be taken into account when evaluating the findings of the present study, it is worth noting that some of the analytes analysed underwent variations similar to those recorded after water intake, after a standardized breakfast intake including coffee, and after mate intake before phlebotomy (*6*, *9*, *12*, *21*). In none of the above-mentioned cases, were the changes higher than the RCV. Based on these data, it urges to re-examine the current patient preparation instructions for routine biochemical laboratory tests as well as to reach a consensus on the type of statistics to be applied to the study of the influence of preanalytical variables on laboratory test results.

Finally, based on our results, it can be concluded that drinking a cup of coffee 1 h prior to phlebotomy produces no clinically significant changes in routine biochemical and haematological test results.
